# Prescribing patterns for attention deficit hyperactivity disorder medications among children and adolescents in Korea, 2007-2011

**DOI:** 10.4178/epih.e2016045

**Published:** 2016-10-26

**Authors:** Inmyung Song, Ju-Young Shin

**Affiliations:** 1 Division of Risk Assessment and International Cooperation, Centers for Disease Control and Prevention, Cheongju, Korea; 2 School of Pharmacy, Sungkyunkwan University, Suwon, Korea

**Keywords:** Attention deficit hyperactivity disorder, Methylphenidate, Atomoxetine hydrochloride, Attention, Prescriptions

## Abstract

**OBJECTIVES:**

This study analyzed the prevalence of attention deficit hyperactivity disorder (ADHD) medication use among children and adolescents in Korea between January 1, 2007 and December 31, 2011.

**METHODS:**

Using the Korea National Health Insurance claims database, we identified patients between one and 17 years of age who had at least one medical claim for the diagnosis of ADHD (International Classification of Diseases, 10th revision: F90.0). The annual prevalence of ADHD diagnoses was calculated, using national census data from Statistics Korea on the population aged between one and 17 years as the denominator. The prevalence was age-standardized using the 2010 population as the standard population. The number of patients who were treated with methylphenidate and/or atomoxetine and the prevalence of total patients with ADHD that were treated with either drug were also calculated for each year. All analyses were stratified according to gender and age group (1-5 years, 6-12 years, and 13-17 years).

**RESULTS:**

The number of patients diagnosed with ADHD increased from 72,704 persons (0.71%) in 2007 to 85,468 persons (0.93%) in 2011. The annual age-standardized prevalence of ADHD diagnoses increased from 0.67% in 2007 to 0.94% in 2011. The prevalence of methylphenidate use among children and adolescents with ADHD decreased from 73.91% in 2007 to 70.33% in 2011, whereas that of atomoxetine use increased from 5.77% in 2009 to 13.09% in 2011.

**CONCLUSIONS:**

While methylphenidate remains the most commonly prescribed ADHD drug, the use of atomoxetine has increased.

## INTRODUCTION

Attention deficit hyperactivity disorder (ADHD) is the most common pediatric mental disorder, and the prevalence of ADHD is increasing in many parts of the world [1]. The use of ADHD medications such as methylphenidate in the US and Europe has been extensively studied [2,3], but in Asia, it has only been studied in Hong Kong [4]. Most studies have reported dramatic increases in the use of ADHD medications.

It has been documented that methylphenidate has greater effects than atomoxetine on performance measures of sustained attention in youths with ADHD [5]. The rate of new atomoxetine use in the US decreased in all age groups even before the Food and Drug Administration issued a warning concerning suicidal thinking [6]. Since the approval of atomoxetine, a newer ADHD medication, in 2006, changes in patterns of prescribing ADHD medications in Korea have not been fully explored. This study aimed to investigate the prevalence of ADHD diagnoses and changing patterns in the prescription of methylphenidate and atomoxetine among children and adolescents with ADHD between January 1, 2007 and December 31, 2011.

## MATERIALS AND METHODS

### Study subjects

This study was conducted using the Korea National Health Insurance (NHI) claims database. The study subjects consisted of children and adolescents in Korea aged between one and 17 years who had been diagnosed with ADHD between January 1, 2007 and December 31, 2011. We identified patients who had at least one medical claim containing an International Classification of Diseases, 10th revision code for ADHD (F90.0) as a primary or secondary diagnosis. The ADHD medications included methylphenidate and atomoxetine prescribed at least once in inpatient or outpatient settings. Methylphenidate and atomoxetine are the only drugs that have been approved for the treatment of ADHD in Korea; dexamphetamine has not been approved. All preparations of methylphenidate, such as immediate release and sustained release formulations, were included.

### Prevalence of attention deficit hyperactivity disorder diagnoses and attention deficit hyperactivity disorder medication use

The annual prevalence of ADHD diagnoses was calculated using the national census data from Statistics Korea on the total population aged between one and 17 years as the denominator. The prevalence of ADHD diagnosis was age-standardized using the 2010 population as the standard population. The number and proportion of patients with ADHD who were treated with either methylphenidate or atomoxetine were also calculated for each year. The number of prescriptions of ADHD medications per year and the prescribed days of ADHD medication per patient were also estimated. All analyses were stratified by gender and age group (1-5 years, 6-12 years, and 13-17 years).

To examine the time trend of prevalence, the monthly numbers of patients diagnosed with ADHD and patients treated with medications were calculated and plotted. Drug treatment groups were categorized into methylphenidate users, atomoxetine users, and combined users.

All statistical analyses were performed using SAS version 9.3 (SAS Institute Inc., Cary, NC, USA). The study protocol was approved by the institutional review board (IRB) of the Sungkyunkwan University (IRB no. 2016-08-013).

## RESULTS

Overall, fewer than 1% of children and adolescents in Korea were diagnosed with ADHD in 2007-2011. The total number of patients diagnosed with ADHD increased from 72,704 persons (0.71%) in 2007 to 85,468 persons (0.93%) in 2011. The age-standardized annual prevalence of ADHD diagnosis increased from 0.67% in 2007 to 0.94% in 2011. The prevalence increased from 1.07% to 1.41% in boys and from 0.30% to 0.41% in girls over the study period. The prevalence increased from 1.09% to 1.45% in children 6-12 years of age and from 0.58% to 0.94% in those 13-17 years of age. The mean (±standard deviation [SD]) age of the diagnosed patients increased from 10.41 (±3.18) years in 2007 to 11.19 (±3.20) years in 2011. The mean age of patients on methylphenidate or atomoxetine increased as well. The number of diagnosed patients aged 1-5 years decreased from 2,944 (0.13%) to 2,449 (0.11%), while the number of patients 13-17 years of age diagnosed with ADHD increased from 20,316 (0.58%) to 31,413 (0.94%) between 2007 and 2011 (Table 1).

**Figure 1.**
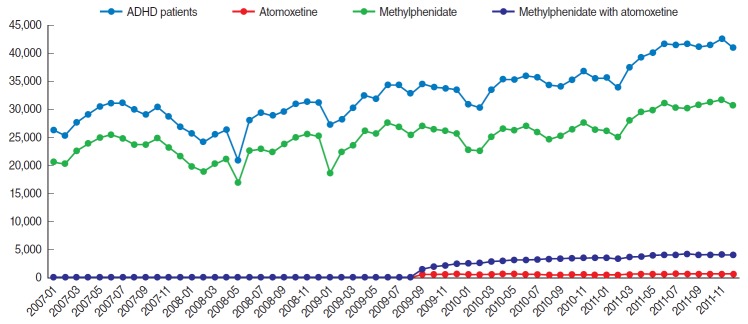
Time trends in attention deficit hyperactivity disorder (ADHD) diagnoses and use of methylphenidate and atomoxetine, 2007-2011.

Moreover, the monthly number of ADHD diagnoses and patients prescribed medication showed generally upward trends, except for atomoxetine use (Figure 1). The majority of patients diagnosed with ADHD and treated with medication were boys. Over 70% of the patients diagnosed with ADHD were prescribed either methylphenidate or atomoxetine. The number of methylphenidate users increased from 53,739 in 2007 to 60,108 in 2011, whereas the number of atomoxetine users increased from 4,267 in 2009 to 11,190 in 2011. The proportion of methylphenidate users among ADHD patients decreased from 73.91% in 2007 to 70.33% in 2011. The proportion decreased from 74.83% to 71.09% in boys and from 70.31% to 67.47% in girls. The proportion of atomoxetine users among ADHD patients increased from 5.77% in 2009 to 13.09% in 2011. This proportion increased from 6.09% to 13.74% for boys and from 4.50% to 10.67% for girls. The proportion of methylphenidate users among patients aged 1-5 years decreased from 27.41% in 2007 to 7.59% in 2011. The number of prescriptions per patient increased from 6.85 in 2007 to 8.59 in 2011 for methylphenidate, while the number increased from 3.63 in 2009 to 6.87 in 2011 for atomoxetine. The number of prescribed days of ADHD medication per patient increased from 143.73 days in 2007 to 177.11 days in 2011 for methylphenidate, whereas it increased from 66.78 days in 2009 to 142.19 days in 2011 for atomoxetine (Table 2).

## DISCUSSION

This study showed that less than 1% of children and adolescents in Korea were diagnosed with ADHD in 2007-2011. A recent study in Korea also showed that the prevalence of ADHD diagnosis and medication use in the pediatric population aged 6-18 years during 2008-2011 was 0.799% and 0.610%, respectively [7]. Current and previous studies suggest that the prevalence of ADHD diagnoses in Korea is low compared with other countries. Among US children and adolescents aged 4 to 17 years, the parent-reported prevalence of ADHD in 2011 was 8.8%, and the prevalence of pharmacologically treated ADHD was 6.1%, with the latter having increased by 28% from 2007 [1]. The prevalence of ADHD diagnosis and medication use in Canadian children was around 3% [8]. The prevalence of ADHD diagnoses in Korea was on par with that in Hong Kong, where the prevalence of children on medication was 1.027% in 2013 [4]. The presence of differences among countries suggests that some children with ADHD are undiagnosed in Korea. This is especially likely given the finding of a 6.5% ADHD prevalence rate in a cross-sectional survey of elementary school children in Seoul [9].

The age-standardized annual prevalence of ADHD diagnoses in Korea increased substantially, from 0.67% in 2007 to 0.94% in 2011. Increasing trends have also been reported in many other countries. The rate of ADHD diagnosis in the US increased from 2.5% in 2001 to 3.1% in 2010 [10]. In the US pediatric population aged 0-17 years, ADHD prescriptions increased by 46% from 2002 to 2010 [2]. The prevalence of pharmacologically treated ADHD in the UK (per 1,000 persons) increased from 4.8 to 9.2 in children 6-12 years of age and from 3.6 to 7.4 in those aged 13-17 years from 2003 to 2008 [3]. The prevalence of children on ADHD medications in Hong Kong saw a 14-fold increase from 2001 to 2013 [4].

We found that the age structure of patients diagnosed with ADHD changed over time, with the mean age of patients becoming older. The number of patients aged 1-5 years decreased, while more patients aged 13-17 years were diagnosed with ADHD. Likewise, increases in the prevalence of ADHD in Canada were steeper among school-age children than among preschoolers between 1994 and 2007 [8].

A gender disparity in the prevalence of ADHD diagnosis and medication use was also noted in this study. Not only was the number of boys diagnosed with ADHD greater than that of girls, but the proportion of ADHD patients prescribed medication was also higher for boys than for girls. This is consistent with previous findings [9]. Nonetheless, our study indicated that the gender disparity decreased, because increases in ADHD prevalence and medication use were steeper among girls. Closing of the gender gap over time has been reported in the US [10] and Canada [8]. Additionally, the use of ADHD medications increased at a faster rate for female patients than for their male peers in the UK [3] and Hong Kong [4].

Along with the increasing prevalence of ADHD diagnoses over time, the number of prescriptions of ADHD medication per year and the number of prescribed days of ADHD medication per patient also increased. A prior study showed that 69% of newly diagnosed ADHD patients in Korea were pharmacologically treated and that methylphenidate was the most commonly used first medication [7]. Our data also indicated that over 70% of pediatric patients diagnosed with ADHD were prescribed methylphenidate, although its use showed a downward trend. The decrease in the number of methylphenidate users aged 1-5 years may be partially attributable to the announcement of the Korean regulatory body that the drug is contraindicated in this age group due to the risk of sudden cardiac death in December 2009 [11]. While the prevalence of methylphenidate users decreased, that of atomoxetine users increased, suggesting that some patients switched from methylphenidate to atomoxetine since the approval of the latter in Korea in 2009. It is worth noting that 6.61% of patients in our analysis used both methylphenidate and atomoxetine in 2010. In comparison, a Danish study showed that only 2% of methylphenidate prescriptions overlapped with atomoxetine treatment [12].

Our data suggest that a seasonal trend was present in ADHD diagnoses and the use of methylphenidate. This seasonal pattern appears to be attributable to school-year dosing in children; the number of diagnoses and methylphenidate users decreased in school-break months in the school calendar in Korea. However, similar seasonal variations were not observed for atomoxetine and combined ADHD drug users. It is plausible that our observed seasonal variation may be associated with the inappropriate prescribing of methylphenidate. Anecdotal experiences and/or perceptions of the incorrect belief that the use of methylphenidate may heighten a child’s learning ability, thus improving test scores, may have contributed to the use of methylphenidate and the increase in ADHD diagnoses required for its prescription during spring and fall semester, followed by discontinuation during school breaks. It is possible that this phenomenon was observed only for methylphenidate, an ADHD drug that has been widely used since the 1990s, but not for atomoxetine, a relatively new drug.

A limitation of this study is that it used data on patients with ADHD obtained from the NHI database. It is highly likely that psychological disorders such as ADHD may be diagnosed and treated outside of the NHI system and paid for with out-of-pocket payments due to the stigma attached with the disorder. Therefore, the prevalence of ADHD diagnoses and medication use might have been underestimated in this study.

Additionally, the study subjects were limited to the population aged 17 years and younger, as most ADHD drugs approved by the Ministry of Food and Drug Safety are indicated for children and adolescents aged 6-17 years. However, as the prevalence of ADHD diagnoses has recently increased in adults and ADHD drug reimbursement has also been expanded to include adults (18-65 years) [13], future research is urgently needed to include all adolescents and adults in such studies using the latest data.

In conclusion, this study revealed that the prevalence of ADHD diagnoses and medication use in Korea increased from 2007 to 2011, although it remained relatively low compared to Western countries. The proportion of methylphenidate users among pediatric patients with ADHD decreased while the proportion of atomoxetine users increased. Future research is needed to examine comparative drug use patterns and how changes in the epidemiology of ADHD medication use affect patient safety.

## Figures and Tables

**Table 1.** Age-standardized annual prevalence of attention deficit hyperactivity disorder (ADHD) diagnoses, 2007-2011^1^

Category	Total patients with ADHD				
	2007	2008	2009	2010	2011
Total (n)	72,704	68,440	73,963	76,308	85,468
Crude prevalence (%)	0.71	0.69	0.76	0.81	0.93
Age-standardized prevalence (%)^2^	0.67	0.67	0.75	0.81	0.94
Gender					
Boys	58,005 (1.07)	54,684 (1.05)	59,160 (1.17)	60,899 (1.24)	67,487 (1.41)
Girls	14,699 (0.30)	13,756 (0.29)	14,803 (0.32)	15,409 (0.34)	17,981 (0.41)
Age (yr)	10.41±3.18	10.69±3.16	10.94±3.14	11.04±3.19	11.19±3.20
Age group (yr)					
1-5	2,944 (0.13)	2,445 (0.11)	2,151 (0.10)	2,215 (0.10)	2,449 (0.11)
6-12	49,444 (1.09)	45,018 (1.09)	47,148 (1.20)	47,631 (1.28)	51,606 (1.45)
13-17	20,316 (0.58)	20,977 (0.59)	24,664 (0.71)	26,462 (0.77)	31,413 (0.94)

Values are presented as number (%) or mean±standard deviation.

1 ADHD prevalence was calculated using Korean census statistics as the denominator.

2 Age-standardized prevalence was estimated using the direct-standardization method with the 2010 standard population.

**Table 2.** Proportion of methylphenidate and atomoxetine prescriptions among patients with ADHD, number of prescriptions, and prescribed days of ADHD medication per patient, 2007-2011^1^

Category	Methylphenidate					Atomoxetine			Atomoxetine + methylphenidate combined		
	2007	2009	2009	2010	2011	2009	2010	2011	2009	2010	2011
Total (n)	53,739	50,386	54,301	53,721	60,108	4,267	9,646	11,190	2,841	5,041	5,188
Medication/diagnosed patients (%)	73.91	73.62	73.42	70.40	70.33	5.77	12.64	13.09	3.84	6.61	6.07
No. of prescriptions	6.85±5.57	8.08±7.15	8.37±7.15	8.48±7.21	8.59±7.1	3.63±2.66	6.63±6.2	6.87±6.31	8.39±7.17	8.80(±7.33)	8.86±7.21
Prescribed days	143.73±111.19	152.57±115.19	163.32±117.41	169.18±119.72	177.11±121.68	66.78±43.31	131,08±110.5	142.19±116.13	165.1±118.7	178.6±124.4	186.2±125.7
Gender											
Boys	43,404 (74.83)	40,728 (74.48)	43,836 (74.10)	43,285 (71.08)	47,976 (71.09)	3,601 (6.09)	8,056 (13.23)	9,272 (13.74)	2,391 (3.23)	4,238 (5.73)	4,354 (5.89)
Girls	10,335 (70.31)	9,658 (70.21)	10,465 (70.70)	10,436 (67.73)	12,132 (67.47)	666 (4.50)	1,590 (10.32)	1,918 (10.67)	450 (0.61)	803 (1.09)	834 (1.13)
Age (yr)	10.71±3.00	10.98±2.96	11.19±2.94	11,35±2.94	11.46±2.96	10.61±2.67	11.01±2.71	11.19±2.80	11.18±2.93	11.34±2.92	11.46±2.94
Age group (yr)											
1-5	807 (27.41)	550 (22.49)	333 (15.48)	185 (8.35)	186 (7.59)	13 (0.60)	6 (0.27)	9 (0.37)	5 (0.01)	4 (0.01)	3 (0.00)
6-12	36,995 (74.82)	33,505 (74.43)	35,147 (74.55)	33,961 (71.30)	37,032 (71.76)	3,217 (6.82)	6,756 (14.18)	7,473 (14.48)	2,189 (2.96)	3,656 (4.79)	3,679 (4.30)
13-17	15,937 (78.44)	16,331 (77.85)	18,821 (76.31)	19,575 (73.97)	22,890 (72.87)	1,037 (4.20)	2,884 (10.90)	3,708 (11.80)	647 (0.87)	1,381 (1.81)	1,506 (1.76)

Values are presented as number (%) or mean±standard deviation.

ADHD, attention deficit hyperactivity disorder.

1 ADHD prevalence was calculated using the Korean census statistics as the denominator.
